# *Coelogyne magnifica* (Orchidaceae), a new species from northern Myanmar

**DOI:** 10.3897/phytokeys.88.19861

**Published:** 2017-10-12

**Authors:** Bin Yang, Shi-Shun Zhou, Qiang Liu, Kyaw Win Maung, Ren Li, Rui-Chang Quan, Yun-Hong Tan

**Affiliations:** 1 Southeast Asia Biodiversity Research Institute, Chinese Academy of Sciences, Yezin, Nay Pyi Taw 05282, Myanmar; 2 Centre for Integrative Conservation, Xishuangbanna Tropical Botanical Garden, Chinese Academy of Sciences, Menglun, Mengla, Yunnan 666303, P.R. China; 3 Gardening and Horticulture Department, Xishuangbanna Tropical Botanical Garden, Chinese Academy of Sciences, Menglun, Mengla, Yunnan 666303, P.R. China; 4 Forest Research Institute, Forest Department, Ministry of Environmental Conservation and Forestry, Yezin, Nay Pyi Taw 05282, Myanmar

**Keywords:** Kachin state, section Ocellatae, key, plant taxonomy, IUCN, Hponkan Razi

## Abstract

*Coelogyne magnifica* (Orchidaceae), a new species from Putao, Kachin State, Myanmar, is described and illustrated. It belongs to Coelogyne section Ocellatae Pfitzer & Kraenzl. and it is morphologically similar to *Coelogyne corymbosa* and *C. taronensis*, but can be distinguished from these species by its larger flowers, lanceolate sepals and petals, a narrowly ovate lip, which has two bright yellow patches surrounded by shiny brownish red and two fimbriate or erose-lacerate lateral keels on the lip. The major differences between these species are outlined and discussed.

## Introduction

The genus *Coelogyne* Lindl. (Lindley 1821), which consists of approximately 200 species, is distributed from South India, through tropical Asia and the Malay Archipelago into the Pacific as far east as Fiji, with the main centres being in Borneo, Sumatra and the Himalayas (Gravendeel 2000, Clayton 2002, Gravendeel et al. 2001, 2005, Chen and Clayton 2009, George and George 2011). It belongs to the subfamily Epidendroideae, tribe Coelogyneae, subtribe Coelogyninae (Gravendeel et al. 2005). As currently circumscribed based on molecular phylogenetic evidence, *Coelogyne* is polyphyletic and composed of species belonging to two unrelated groups. It would thus be questionable whether to adopt a *Coelogyne* *s.s.* generic delimitation or a *Coelogyne* *s.l.* generic circumscription (Gravendeel 2000, Gravendeel et al. 2001). Although revisions of several sections of *Coelogyne* have been published in the last decade (Gravendeel and de Vogel 1999, Pelser et al. 2000), a comprehensive infrageneric delimitation combined with descriptions of morphological and molecular characters based on more extensive sampling within *Coelogyne* is needed (Gravendeel et al. 2000, Sierra et al. 2000).

Historically, the study of *Coelogyne* in Myanmar dates back to Hooker (1890), who reported three *Coelogyne* species and added one new species *Coelogyne longibractata* Hook. f.. Currently, 43 species of *Coelogyne* have been recorded in Myanmar (Kress et al. 2003), although in a recently published field guide to the orchids of Myanmar, only 33 *Coelogyne* species are described (Kurzweil and Lwin 2014). The most recently identified new species of *Coelogyne* was *Coelogyne putaoensis* X.H. Jin, L.A. Ye & Schuit. is from north Myanmar (Aung et al. 2017). Since the publication of Kress’s checklist (Kress et al. 2003), more than 10 new species and 40 new records of Orchidaceae species have been added to the flora of Myanmar from 2001 to the present (Yang and Tan, unpublished data).

During recent China–Myanmar joint field expeditions to survey plant diversity in north Myanmar in May 2016 and 2017, specimens of *Coelogyne* were found in Putao, Kachin State. On the basis of a detailed examination of the morphological and anatomical characters of this material and of presumed closely similar species (Clayton 2002, Kress et al. 2003, Chen and Clayton 2009, George and George 2011, Subedi 2011, Yonzone 2012a, 2012b, Li and Dao 2014, Gogoi et al. 2015, Aung et al. 2017), the conclusion was made that the specimens collected in Myanmar belong to a species new to science, which is herein described and illustrated.

## Material and methods

Measurements and morphological character assessments of the putative new *Coelogyne* species were performed and described using specimens and fresh material observed in the field. These data were compared with those for the morphologically similar species *C. corymbosa* Lindl. and *C. taronensis* Hand.-Mazz. based on the descriptions of dried herbarium specimens deposited at Xishuangbanna Tropical Botanical Garden herbarium (HITBC), field notes (for *C. corymbosa* which has also been collected from north Myanmar) and literature descriptions (Chen and Clayton 2009, Subedi 2011, Li and Dao 2014). Protologues and images of type specimens were obtained from Tropicos (http://www.tropicos.org), JSTOR Global Plants (http://plants.jstor.org) and the International Plant Names Index (http://www. ipni.org).

## Taxonomic treatment

### Coelogyne magnifica

Taxon classificationPlantaeAsparagalesOrchidaceae

Y.H. Tan, S.S. Zhou & B. Yang sp. nov.

urn:lsid:ipni.org:names:60475278-2

Figures 1 , 2

#### Diagnosis.

*Coelogyne magnifica* is similar to *C. corymbosa* and *C. taronensis*, but can be distinguished from these two species by its larger flowers (tepals 4.0–4.9 cm long), broadly lanceolate sepals and petals, narrowly ovate lip, which has two bright yellow patches surrounded by shiny brownish red and two fimbriate or erose-lacerate keels on the lip.

#### Type.

MYANMAR. Kachin State: Putao, Hponkan Razi Wildlife Sanctuary, 96°58'56.45"E, 27°36'32.42"N, alt. 2450 m, 11 May 2017, *Myanmar Exped. 2046* (holotype, HITBC).

#### Description.

Epiphytic or lithophytic herb, 11–15 cm tall. Rhizome stout, 3–4 mm in diameter, covered by brown, scaly sheaths. Pseudobulbs clustered, developing serially, less than 1 cm apart from each other, ovoid or oblong-ovoid, strongly wrinkled when dried, 3–3.5 × 1.1–1.7 cm, covered with brown sheaths at the base, sheaths narrow triangular ovate, 1.5–4.5 × 0.7–1.5 cm long; bifoliate at apex. Leaf blade obovate-oblong or narrowly ovate, coriaceous, 8–11.2 × 1.8–2.6 cm, with 6–7 veins, acute; petiole grooved, 0.4–0.8 cm long. Inflorescence proteranthous to synanthous, peduncle arching, 5–6 cm long, embraced by sheaths below middle, rachis 1.5–2 cm long; raceme 2- to 3-flowered (simultaneously opening); floral bracts oblong-lanceolate, 1.9–2.1 × 0.4–0.5 cm, acute, caducous. Flowers fleshy, white, lip adaxially on mid-lobe with two bright yellow patches surrounded by shiny brownish red and connected to front part of each of the side lobes, keels consisting of two parallel crests with white fimbriate prominence. Pedicel and ovary ca. 1.8–2.5 cm long, glabrous. Dorsal sepal lanceolate, 4.0–6.0 × 1.0–1.3 cm, with 7–9 veins, acute or shortly acuminate; lateral sepals similar to dorsal sepal, somewhat narrower than dorsal sepal, 5.0–5.5 × 0.9–1.5 cm, with 5-7 veins, acute or shortly acuminate; petals lanceolate, 4.0–5.3 × 0.6–1.0 cm, acute or shortly acuminate; lip narrowly ovate when flattened, 3.8–4.9 × 1.7–2.2 cm, 3-lobed; lateral lobes erect, sub-orbicular, 2.0–2.5 × 0.7–1.0 cm, fimbriate or erose-lacerate on margin, adaxially with reddish brown longitudinal stripe; median lobe ovate-triangular or triangular-lanceolate, 1.8–2.5 × 1.0–1.2 cm, acute to apiculate, margin fimbriate or erose-lacerate; two low lateral keels, extending from lip base to base of median lobe, margin fimbriate or erose-lacerate; column arcuate, 2.0–2.5 × 0.4–0.6 cm long, yellow at front below apex, both sides winged, wings gradually broadening from the middle to the apex, apex margin white, irregularly toothed; anther cap elliptic triangular, adaxially light pale yellow-green, abaxially white, margin membranous at base, apex obtuse; pollinia two; rostellum ligulate-triangular.

**Figure 1.**
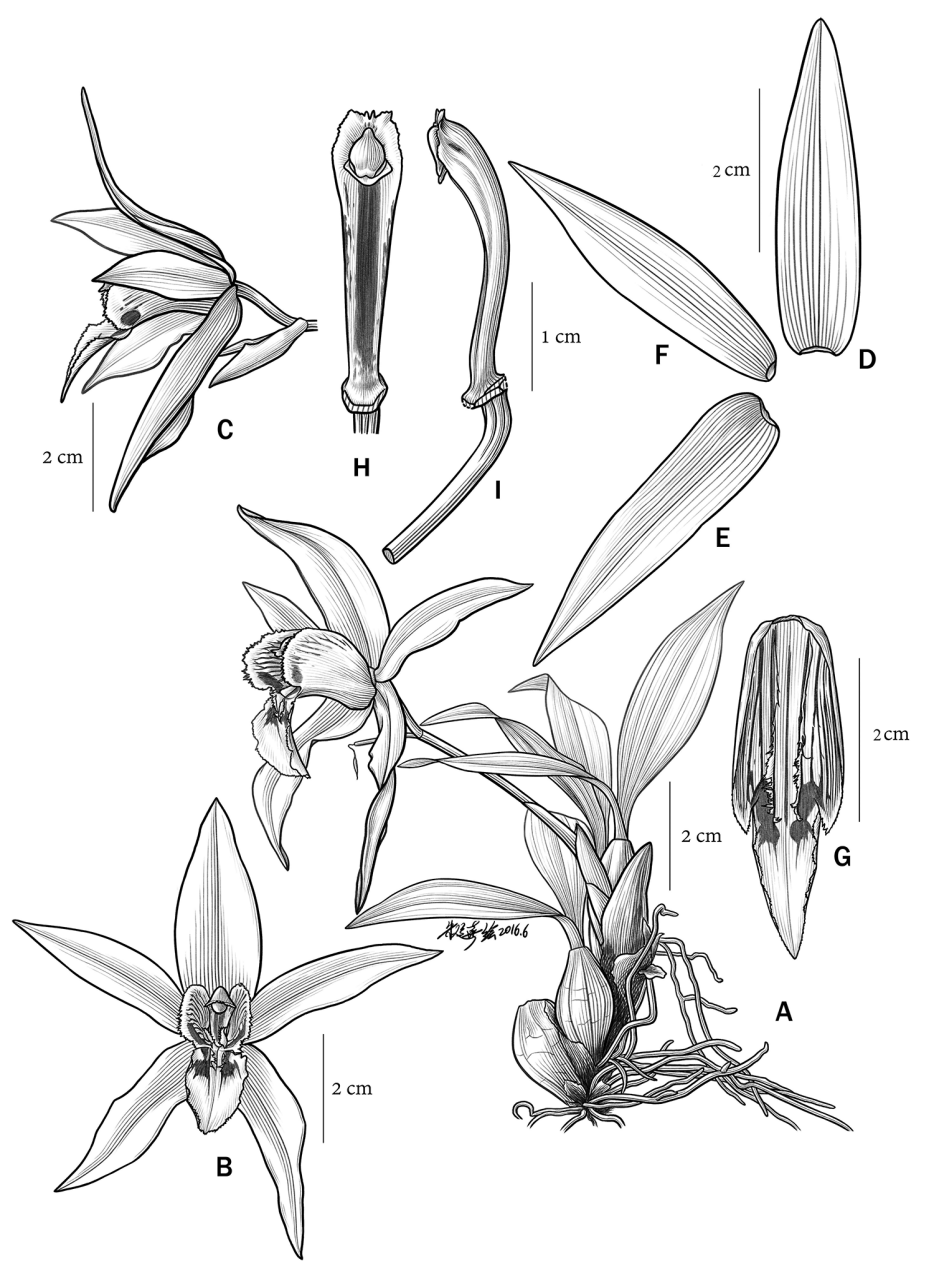
*Coelogyne magnifica* Y.H. Tan, S.S. Zhou & B. Yang sp. nov. **A** Habit **B** Flower (front view) **C** Flower (side view) **D** Dorsal sepal **E** Lateral sepal **F** Petal **G** Lip **H** Column (frontal view) **I** Column (lateral view). Illustration by Yunxi Zhu.

**Figure 2.**
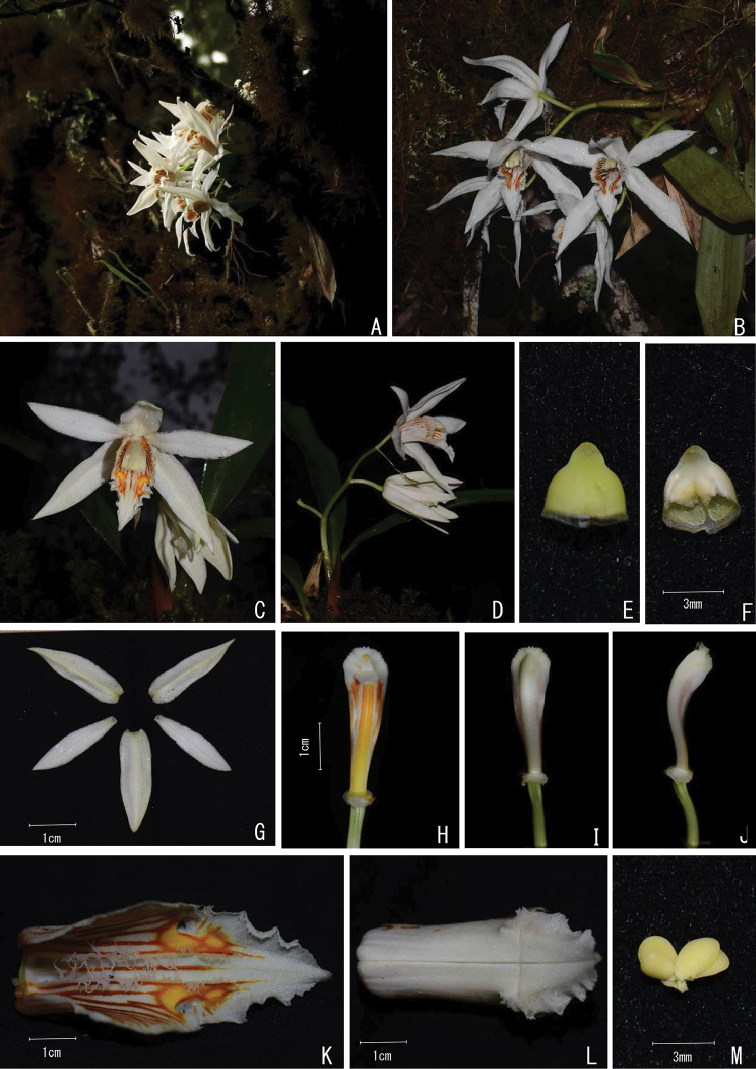
*Coelogyne magnifica* Y.H. Tan, S.S. Zhou & B. Yang sp. nov. **A–D** Habit **E** Anther cap (abaxial view) **F** Anther cap (adaxial view) **G** Tepals **H** Column (adaxial view) **I** Column (abaxial view) **J** Column (lateral view) **K** Lip (adaxial view, showing the two lateral keels and two patches) **L** Lip (abaxial view) **M** Pollinia. Photographed by Y.H. Tan, Q. Liu & X.L. Zeng.

#### Phenology.

Flowering from April to May and fruiting from June to July.

#### Etymology.

The species epithet refers to its large attractive flowers.

#### Distribution and habitat.

*Coelogyne magnifica* is currently known only from the type locality of Putao, Kachin State, northern Myanmar. It is a predominantly epiphytic species that grows on moss-covered branches and tree trunks and sometimes also on rocks, in humid montane forests, at an elevation 2400–2500 m a.s.l.

#### Conservation status.

The type locality of this new species is within the Hponkanrazi Wildlife Sanctuary, which is an officially protected area under the management of the Myanmar Forest Department. During field investigations in 2016 and 2017, three populations were found in the reserve area, each of which consisted of ca. 100 individuals. As found, inhabitants are well protected and almost undamaged and flowering individuals are not vulnerable to human interference or picking. Based on current information and according to IUCN Red List category (IUCN 2012), *Coelogyne magnifica* is assigned a preliminary status of Least Concern (LC).

#### Additional specimens examined (paratypes).

Myanmar. Kachin State: Putao, Hponkanrazi Wildlife Sanctuary, 97°53'10.48"E, 27°41'17.60"N, alt. 2400 m, 4 May 2016, *Myanmar Exped. 0100* (HITBC). Ibid., alt. 2450 m, 7 May 2016, *Myanmar Exped. 0188* (HITBC).

#### Key to the species of Coelogyne sect. Ocellatae, adapted from Li and Dao (2014)

**Table:** 

1	Inflorescence hysteranthous	**2**
–	Inflorescence proteranthous or synanthous	**3**
2	Lowermost bract of rachis with flower	***Coelogyne hysterantha***
–	Lowermost bract of rachis sterile	***Coelogyne punctulata***
3	Pseudobulb obovoid, born distantly, 1.3–5.0 cm apart	***Coelogyne occultata***
–	Pseudobulb ellipsoid, born clustered, less than 1.3 cm apart	**4**
4	Keels on lip plate-like shaped	***Coelogyne platylamellata***
–	Keels on lip clavate or rod-shaped	**5**
5	Keels starting 0.3 cm away from the base of the hypochile	***Coelogyne gongshanensis***
–	Keels starting right from the base of the hypochile	**6**
6	Lateral lobes of lip length 3–3.2 cm when flattened	***Coelogyne wardii***
–	Lateral lobes of lip length 1–2.5 cm when flattened	**7**
7	Margin of lateral lobes of lip crenulate or crispate	**8**
–	Margin of lateral lobes of lip erose or denticulate	**9**
8	Flowers almost solid yellowish brown, lip with a large, bright yellow patch	***C. putaoensis***
–	Flowers creamy-yellow, lip with four dark red patches	***C. taronensis***
9	Lateral keels of lip clavate and with erose lacerate or crenulate margins	**10**
–	Lateral keels of lip rod-shaped and with entire margins	**11**
10	Sepals and petals more than 4.0 cm long	***C. magnifica***
–	Sepals and petals less than 3.5 cm long	**12**
11	Flowers 4–8, mid-lobe acute at apex	***Coelogyne nitida***
–	Flowers 2–3, mid-lobe cuspidate at apex	***Coelogyne ttyuii***
12	Yellow eyelike blotches surrounded by reddish orange on lip	***C. corymbosa***
–	Dark brown eyelike blotches surrounded by brownish-yellow on lip	***Coelogyne pianmaensis***


## Discussion

According to monographic works (Clayton 2002, Chen and Clayton 2009, George and George 2011, Subedi 2011), *Coelogyne magnifica* obviously belongs to sect. Ocellatae Pfitzer & Kraenzl. (Pfitzer and Kraenzlin 1907), which is characterised by the white or pale coloured flowers with colourful eyelike blotches on the lateral lobes and lip. This section shows its centre of diversity in the Himalayas (Subedi 2011). *Coelogyne magnifica* is similar to both *C. corymbosa* and *C. taronensis* in terms of vegetative morphology and shape of the flowers, but differs mainly with respect to the characters of patches and keels on the lip. Morphologically, the new species is most similar to *C. corymbosa*, with both having white flowers, ovoid pseudobulbs sometimes and obovate-oblong leaf blades. These two species are also distributed in the same locality in north Myanmar. Nevertheless, the new species differs from *C. corymbosa* in having slightly smaller pseudobulbs, shorter leaf blades, a greater number of larger flowers, two bright yellow patches surrounded by shiny brownish red (vs. four yellow eye-like blotches) and two fimbriate or erose-lacerate lateral keels on the lip (vs. two–three keels, with margins wavy or crenulate) (Table 1). The tepals (perianth lobes, including sepals, petals and lip) of *C. magnifica* are lanceolate, 4.0–4.9 cm long (vs. mostly elliptic to ovate, 2.4–3.7 cm long in *C. corymbosa*). Although the other morphologically similar species, *C. taronensis*, is similar to the new species with respect to ovoid pseudobulbs and in flower size, the new species differs from *C. taronensis* with regards to its white flowers (vs. creamy yellow in *C. taronensis*) (Figure 2), smaller leaf blades (Table 1), patches (two bright yellow patches vs. four dark red patches) and keels (two keels vs. three keels) on the lip. The major differences between the species are outlined in Table 1.

**Table 1.** Morphological comparison of *Coelogyne magnifica* and its closely related species.

Characters	*Coelogyne magnifica*	*Coelogyne corymbosa*	*Coelogeny taronensis*
Pseudobulbs	ovoid or oblong-ovoid, 2–2.5 × 1–1.3 cm	oblong-ellipsoid to ovoid, 2–6 × 1.1–2.5 cm	ovoid, 2–2.5 × 1.3–1.8 cm
Leaf blade	obovate-oblong or narrowly ovate, 4–6 × 0.8–1.4 cm, 5–7 veined	oblong-oblanceolate to obovate-oblong, 4.5–15 × 1–3 cm	elliptic or obovate, 9.8–13.5 × 2.2–2.3 cm
Pedicel and ovary	1.3–1.6 cm	2.5–3.1 cm	2.9–3.2 cm
Flowers	white	white	creamy-yellow
Dorsal sepal	lanceolate, 4.0–6.0 × 1.0–1.3 cm	elliptic to narrowly ovate, 2.6–3.5 × 0.8–1.3 cm	elliptic to narrowly ovate, 3–3.5 × 1.3–1.7 cm
Lateral sepals	lanceolate, 5.0–5.5 × 0.9–1.5 cm	oblong-elliptic or narrowly ovate, 2.6–3.7 × 0.7–2.1 cm	oblong-elliptic or narrowly ovate, 3.2–3.5 × 0.8–1.2 cm
Petals	lanceolate, 4.0–5.3 × 0.6–1.0 cm	narrowly elliptic or narrowly obovate, 2.4–3.2 × 0.6–1.2 cm	elliptic-oblong, 2.8–3.5 × 0.6–0.8 cm
Lip	narrowly ovate, 3.8–4.9 × 1.7–2.2 cm	ovate or elliptic, 2.1–3.3 × 1.4–2.1 cm	ovate, 2.8–3.9 × 2.1–2.6 cm
Patches or blotches	two bright yellow patches surrounded by shiny brownish red	four yellow blotches surrounded by reddish orange	four dark red patches
Keels	2 fimbriate or erose lacerate	2–3 wavy, entire or crenulate	3 crenulate to wavy and slightly papillose

## Supplementary Material

XML Treatment for Coelogyne magnifica
